# Beyond Nutrient Composition: AGEs and Food Neo-Contaminants in Ultra-Processed Food Toxicity

**DOI:** 10.3390/nu18213621

**Published:** 2026-10-31

**Authors:** Ma. Eugenia Garay-Sevilla, Franco Pedreschi, Jaime Uribarri

**Affiliations:** 1 Departamento de Ciencias Médicas, División Ciencias de la Salud, Universidad de Guanajuato, Campus León, León 37320, Mexico; marugaray_2000@yahoo.com; 2 Departamento de Ingeniería Química y Bioprocesos, Pontificia Universidad Católica de Chile, Santiago 6904411, Chile; fpedreschi@uc.cl; 3 Renal Division, Department of Medicine, Icahn School of Medicine at Mount Sinai, New York, NY 10029, USA

**Keywords:** AGEs, neo-contaminants, UPF, cardiometabolic complications

## Abstract

Worldwide consumption of ultra-processed foods (UPFs) is increasing in parallel with a rising incidence of obesity, type 2 diabetes, cardiovascular disease, kidney disease, and related cardiometabolic complications. Among the many features of UPFs that may drive these outcomes, industrial thermal processing is a defining characteristic that promotes the formation of advanced glycation end products (AGEs) and other food neo-contaminants, including acrylamide, furan, 5-hydroxymethylfurfural, heterocyclic amines, polycyclic aromatic hydrocarbons, 3-monochloropropane-1,2-diol, and glycidol. This review evaluates the hypothesis that AGEs and thermally generated neo-contaminants may contribute to the adverse metabolic and toxicological effects associated with UPFs. After formation in food, these compounds may be partially absorbed in the gastrointestinal tract and act systemically, promoting tissue injury through protein cross-linking, oxidative stress, inflammation, endothelial dysfunction, and activation of the receptor for advanced glycation end products (RAGE). Several high-temperature neo-contaminants are linked to carcinogenicity, hepatotoxicity, neurotoxicity, and reproductive toxicity, while emerging evidence also implicates them in insulin resistance, adipogenesis, β-cell dysfunction, and cardiovascular risk. Overall, the evidence supports a biologically plausible link between thermal processing, AGE and neo-contaminant burden, and cardiometabolic disease, although the strength of evidence varies by compound. Improved exposure assessment standardized analytical methods, mechanistic studies, and intervention trials are needed to define dose–response relationships and to determine whether reformulation and lower-temperature, moisture-rich cooking can reduce risk while maintaining food safety and acceptability.

## 1. Introduction

The incidence of diabetes and its cardiovascular, kidney, and metabolic complications—collectively recognized as cardiovascular–kidney–metabolic (CKM) syndrome—has risen markedly in recent years, paralleling a substantial increase in ultra-processed food (UPF) consumption [1,2,3]. Current data indicate that the United States and the United Kingdom have the highest levels of UPF intake, with such foods accounting for more than half of daily caloric intake; by contrast, countries with more traditional dietary patterns, such as Romania and Colombia, show some of the lowest levels of UPF consumption [4]. Despite strong epidemiological associations between UPF intake and adverse health outcomes, the specific factor or factors responsible for these harmful effects remain incompletely characterized.

A defining feature of many UPFs is the large-scale industrial use of thermal processing to enhance flavor, texture, microbiological safety, and shelf life. Although heat processing improves palatability, UPFs are also associated with increased appetite, excess energy intake, and the development of obesity [5]. Moreover, high-temperature processing creates conditions that favor the formation of advanced glycation end products (AGEs) and other neo-contaminants, including acrylamide, furan, 5-hydroxymethylfurfural, lipid oxidation products, and heterocyclic amines [6].

It should be emphasized, however, that ultra-processing and high-temperature processing are overlapping but not equivalent concepts. Under the NOVA classification, UPFs are defined mainly by their formulation (industrial ingredients and additives seldom or never used in home cooking, such as protein isolates, modified starches, hydrogenated or interesterified oils, flavor enhancers, emulsifiers, and non-nutritive sweeteners) and by the industrial processes used to assemble them, rather than by the intensity of heating [7]; the scheme has also been criticized for classifying foods by their degree of processing rather than by their nutritional and health effects [8]. Some UPFs undergo only mild thermal treatment, whereas minimally processed or home-cooked foods such as grilled or barbecued meats, home-fried potatoes, toasted bread, or roasted coffee can contain substantial amounts of AGEs, acrylamide, heterocyclic amines, or polycyclic aromatic hydrocarbons [8,9]. Processing-derived contaminants are therefore not specific to UPFs. Nevertheless, many widely consumed UPF categories (fried snacks, breakfast cereals, biscuits, processed meats, and products based on refined oils) combine formulations rich in reactive precursors (reducing sugars, free asparagine, proteins, and refined fats) with high-temperature, low-moisture operations such as frying, baking, extrusion, roasting, and oil refining, which favor the formation of these compounds. In this review, AGEs and neo-contaminants are therefore considered as one potential pathway, linked to thermal processing, whether industrial or domestic, through which part of the health burden associated with UPF consumption may arise, acting together with, rather than independently of, formulation, energy density, additives, and overall dietary patterns.

This review explores the hypothesis that AGEs and other thermally generated neo-contaminants may be relevant contributors to the adverse metabolic effects associated with UPF consumption, acting through pathways that promote oxidative stress, inflammation, insulin resistance, and tissue dysfunction. We stress that this is a biologically plausible hypothesis rather than an established dominant mechanism: other attributes of UPFs, including high energy density, poor nutritional quality, disruption of the food matrix, emulsifiers, non-nutritive sweeteners, and contaminants migrating from packaging such as bisphenols, phthalates, and microplastics, are also likely to contribute [8]. Their relative weight, and indeed whether the associations between UPF consumption and chronic disease are causal, remain to be established [8]. Throughout this review, the term “toxicity” is used in a broad sense to denote adverse biological effects relevant to cardiometabolic disease (oxidative stress, inflammation, insulin resistance, endothelial dysfunction, and tissue injury), in addition to the classical toxicological endpoints (genotoxicity, carcinogenicity, hepatotoxicity, neurotoxicity, and reproductive toxicity) established for specific neo-contaminants. We integrate current evidence on the formation, dietary exposure, and biological effects of these compounds and critically evaluate the strength of the evidence linking them to metabolic disease risk.

This narrative review is based on a literature search conducted in PubMed/MEDLINE, Scopus, and Web of Science, complemented by Google Scholar and by the scientific opinions of food safety agencies (mainly the European Food Safety Authority, EFSA). The search covered publications from January 2000 to September 2026, with emphasis on the last ten years and some references from previous years, and combined terms such as “advanced glycation end products”, “carboxymethyllysine”, “methylglyoxal”, “dietary AGEs”, “ultra-processed foods”, “Maillard reaction”, “acrylamide”, “furan”, “5-hydroxymethylfurfural”, “heterocyclic amines”, “polycyclic aromatic hydrocarbons”, “3-MCPD”, “glycidyl esters”, “neo-contaminants”, “insulin resistance”, “cardiometabolic”, and “mitigation”. Original studies (in vitro, animal, and human), systematic and narrative reviews, and risk assessments published in English were considered and selected according to their relevance to the formation of these compounds in foods, human exposure, mechanisms of toxicity, and mitigation strategies; the reference lists of relevant articles were also screened.

## 2. Dietary AGEs

### 2.1. Formation of AGEs in Food

**Figure 1:**
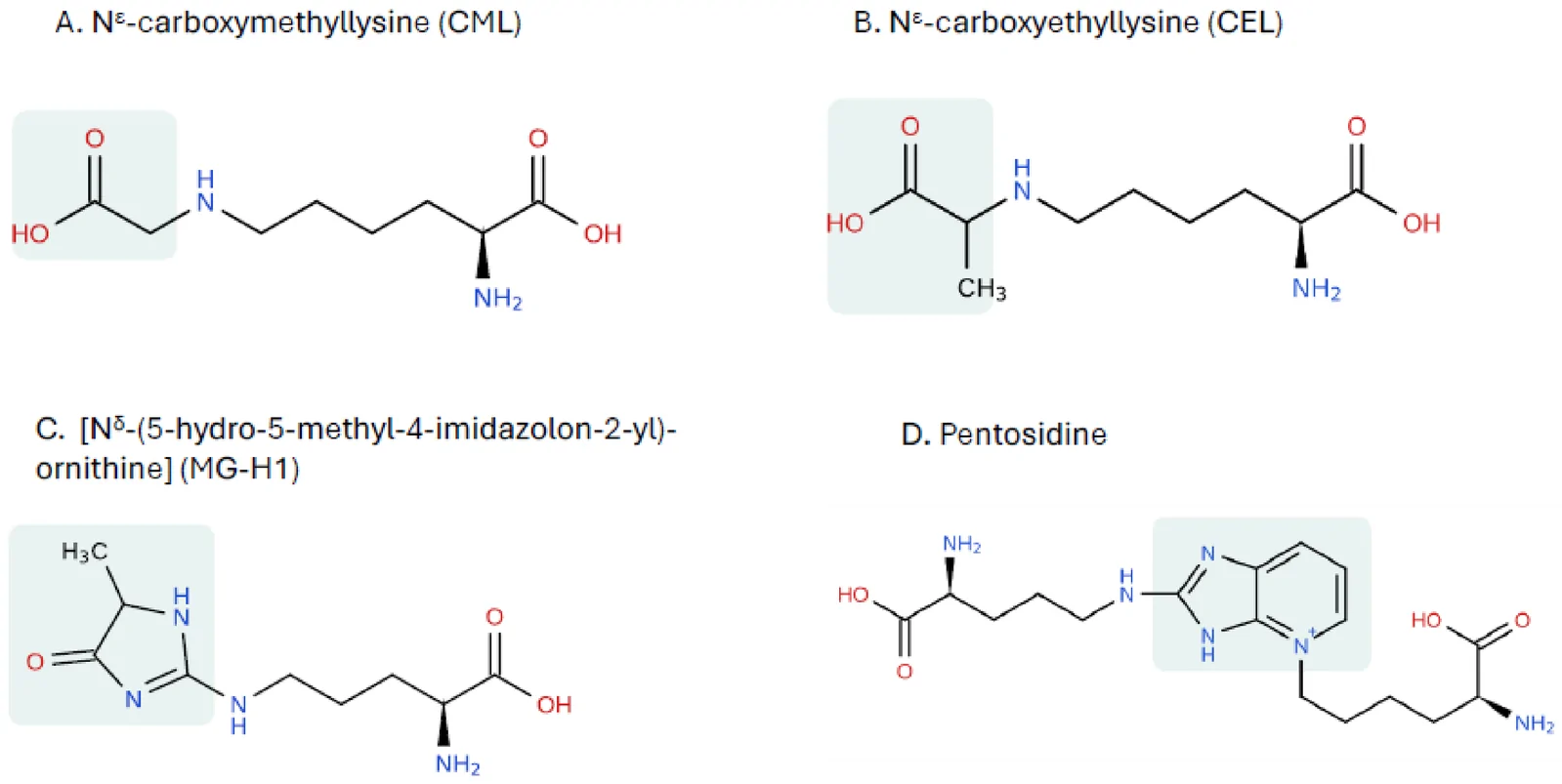
Chemical structures of four well-characterized advanced glycation end products (AGEs): (**A**) N^ε^-carboxymethyllysine (CML); (**B**) N^ε^-carboxyethyllysine (CEL); (**C**) the methylglyoxal-derived hydroimidazolone MG-H1 [N^δ^-(5-hydro-5-methyl-4-imidazolon-2-yl)-ornithine]; and (**D**) pentosidine, a fluorescent lysine–arginine cross-link. Light-blue shaded areas indicate the moiety formed by glycation.

AGEs arise mainly through the Maillard reaction, a non-enzymatic reaction between free amino groups and the carbonyl groups of reducing sugars; they also form through oxidative reactions involving sugars, lipids, and amino acids. Commonly measured and well-characterized AGEs include N^ε^-carboxymethyllysine (CML), N^ε^-carboxyethyllysine (CEL), and the methylglyoxal-derived MG-H1 [N^δ^-(5-hydro-5-methyl-4-imidazolon-2-yl)-ornithine] and pentosidine (Figure 1) [6].

CML is a prototypical AGE and one of the earliest identified glycoxidation products. It forms primarily when lysine residues react with glyoxal (GO), a highly reactive α-dicarbonyl generated during oxidative degradation of reducing sugars, advanced Maillard reactions, and autoxidation of Amadori products. Under high-temperature, low-moisture conditions, the ε-amino group of lysine reacts with glyoxal to produce stable CML adducts [10]. CML is also considered an advanced lipoxidation end product (ALE), reflecting its formation through lipid-derived pathways: peroxidation of polyunsaturated fatty acids generates reactive aldehydes, including glyoxal, which can produce CML independently of sugar availability. This pathway is especially relevant in fat-rich and ultra-processed foods, where lipid oxidation and Maillard chemistry often occur together [11]. Under glycoxidative conditions, Amadori products undergo metal-catalyzed oxidation, generating reactive dicarbonyls that further accelerate CML formation; this process links oxidative stress to AGE accumulation and helps explain why oxygen exposure during food processing and storage promotes CML generation [12].

CEL, another well-characterized AGE, is structurally related to CML but differs in precursor chemistry: it forms through the reaction of methylglyoxal (MGO) with the ε-amino group of lysine. Because MGO is more reactive than glyoxal, CEL formation is particularly relevant in carbohydrate-rich, thermally processed foods [13]. Methylglyoxal promotes post-translational modification of peptides and proteins, ultimately leading to AGEs including the arginine-derived hydroimidazolone MG-H1 and CEL [14]. CEL forms both in biological systems (in vivo) and during food processing (ex vivo). In the body, CEL generation is closely linked to intermediary metabolism, particularly under hyperglycemia or increased glycolytic flux, where MGO production is elevated—a process especially relevant in metabolic diseases such as diabetes mellitus, in which AGE accumulation contributes to tissue damage and chronic inflammation [15]. In processed foods, CEL formation also results from advanced Maillard reactions favored by high temperature and low moisture; during heating, degradation of sugars and lipids generates dicarbonyl compounds, including MGO, which react with food proteins to yield CEL and other AGEs [16]. Oxidative degradation of unsaturated fatty acids likewise generates MGO and related carbonyl species, reinforcing the interplay between Maillard chemistry and lipid oxidation in complex food systems; in experimental model systems, oxidized lipids markedly enhance CEL formation in protein-rich matrices [11].

### 2.2. Dietary Exposure to AGEs

Quantifying human exposure to dietary AGEs has advanced substantially over the past few decades, driven by the development of food composition databases and improvements in analytical chemistry. A foundational resource is the Uribarri et al. database [17], which catalogued AGE content in more than 500 foods prepared using diverse cooking methods. This database has enabled estimation of dietary AGE intake in both clinical and epidemiological studies and has been widely used to link high AGE intake to oxidative stress, inflammation, diabetes, and cardiovascular disease.

Despite its influence, the Uribarri database has important methodological constraints. AGE quantification relied primarily on immunochemical assays—particularly ELISA targeting CML—which capture only a narrow subset of the chemically diverse AGE family. Because AGEs encompass dozens of structurally distinct compounds formed through Maillard chemistry, lipid oxidation, and sugar degradation, reliance on a single marker likely underestimates total dietary exposure. Additional variability arises from differences in food preparation, moisture content, and thermal conditions, which can dramatically alter AGE formation yet are difficult to standardize across studies. The lack of standardization across analytical techniques, variability in preparation conditions, and the inherent difficulty of translating experimental measurements into real-world exposure estimates remain major challenges.

Recent advances have begun to address these limitations. The Glycation Database developed at Abertay University compiles thousands of analytically measured glycation compounds—including early Maillard intermediates, reactive dicarbonyls, and advanced AGEs—across a wide range of foods [18]. Parallel improvements in LC–MS/MS have markedly increased sensitivity and molecular specificity, enabling quantification of individual AGEs such as CML, CEL, and MG-H1, as well as their precursors. These technologies also allow examination of how processing variables—temperature, duration, pH, water activity, and oxygen exposure—influence AGE formation in real food systems [19].

Together, these developments highlight both progress and persistent uncertainty in estimating dietary AGE exposure. Although modern databases and mass spectrometry-based methods offer improved resolution, substantial challenges remain because of the complexity of food matrices and the dynamic nature of industrial processing. Consequently, dietary AGE estimates should be interpreted cautiously, particularly in epidemiological studies where exposure misclassification may obscure true associations with metabolic disease.

### 2.3. Transit of Food AGEs Through the Gastrointestinal Tract

Only a small fraction of food-derived AGEs is absorbed in the small intestine and reaches the circulation. Unabsorbed AGEs pass into the colon, where they may still exert systemic effects—either directly, by acting on the colonic wall to induce local inflammation, or indirectly, by interacting with the colonic microbiota to yield degradation products with or without harmful effects [6]. Initial investigations reported that, following oral intake, approximately 10–30% of dietary AGEs reach the systemic circulation [20,21]. These compounds may be present either as free molecules, including individual amino acids or low-molecular-weight (LMW; <5 kDa) peptides, or attached to proteins as high-molecular-weight (HMW) structures [12]. Free N^ε^-carboxymethyllysine (CML) appears to cross the intestinal barrier through passive diffusion mechanisms [22]. In contrast, pyrraline is predominantly absorbed in its dipeptide form rather than as a free amino acid [23], and its uptake across the intestinal epithelium is mediated by peptide transporter 1 (PEPT1) [24]. Furthermore, the transport of peptide-bound pyrraline across Caco-2 cells was shown to be 15-fold higher compared with free pyrraline [23]. Subsequent studies have demonstrated that PEPT1 is likewise involved in the intestinal transport of dipeptide-bound forms of other AGEs, including CML, CEL, and MG-H1 [25].

Increasing evidence suggests that gut microbiota is a key determinant of the metabolism and physiological impact of dietary AGEs. AGEs that escape absorption in the small intestine and reach the colon may undergo microbial degradation by specific bacterial populations, thereby limiting their systemic exposure [26]. At the same time, prolonged intake of AGE-rich foods has been linked to unfavorable changes in gut microbial composition, reduced production of beneficial short-chain fatty acids, disruption of intestinal barrier function, and enhanced gut permeability. Consequently, the biological and health effects of dietary AGEs appear to depend not only on the amount ingested and absorbed but also on the structure and metabolic capacity of the intestinal microbiota [26,27]. The influence of dietary AGEs on the colonic microbiota is an area of increasing interest [28].

### 2.4. Mechanisms of AGE-Induced Toxicity

**Figure 2:**
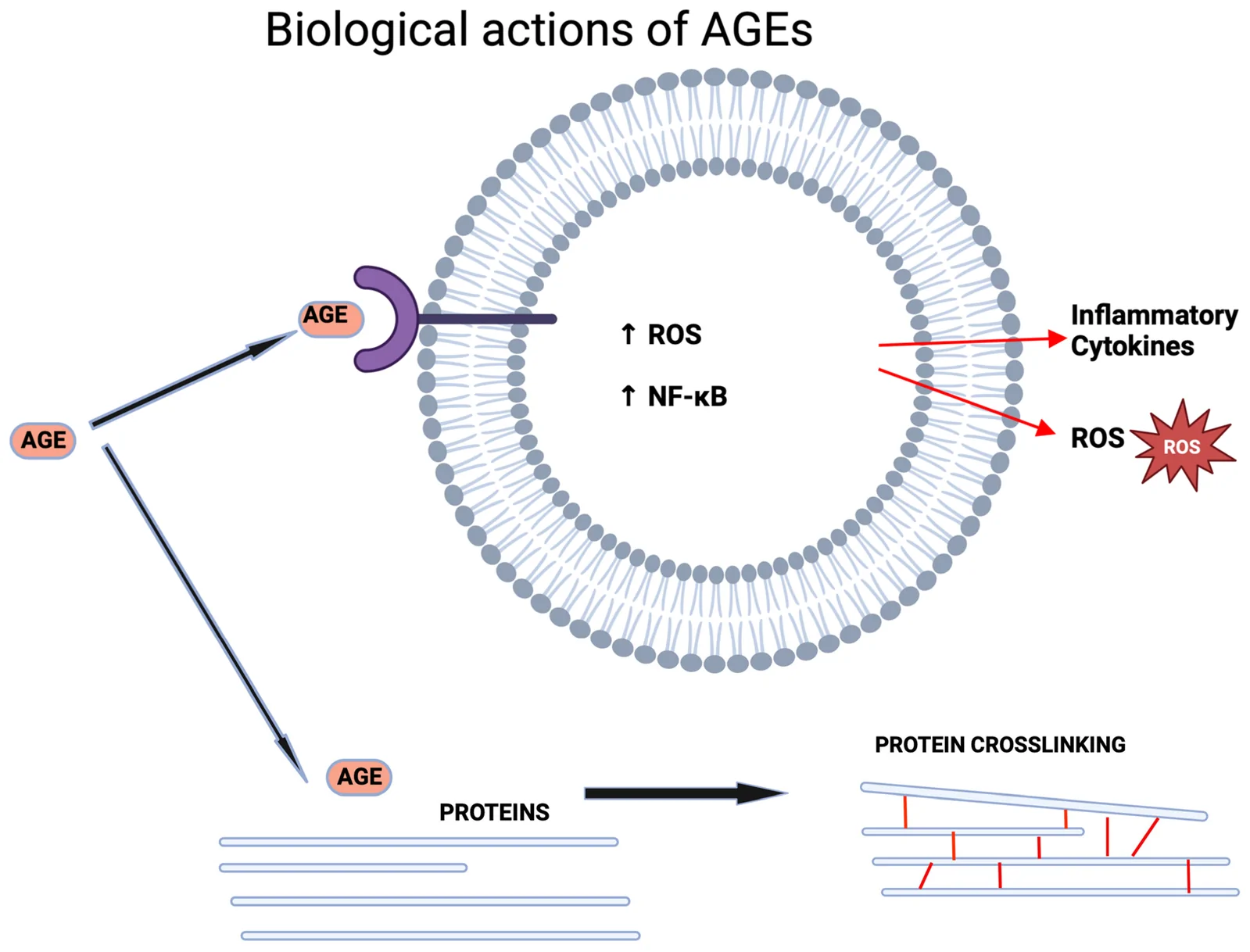
Biological actions of AGEs. Figure 2 illustrates the two main biological actions of Advanced Glycation End Products (AGEs). AGEs, represented as orange ovals, can follow two pathways, either binding to receptors on the cell membrane (RAGE) or modifying directly proteins leading to crosslinking and loss of normal protein function. The upper arrow shows binding to RAGE (purple structure) that initiates intracellular signaling, activating NADPH oxidase, which increases the production of ROS (reactive oxygen species), and also activating NF-κB, a key transcription factor involved in inflammation. NF-κB, in turn, stimulates the expression of pro-inflammatory genes, leading to the production of inflammatory cytokines. The release of inflammatory cytokines and ROS from the cell, which may act locally or systemically, can initiate a vicious cycle of inflammation and oxidative stress. Protein crosslinking is shown here modifying proteins outside the cell, but AGEs can also modify intracellular proteins.

Dietary AGEs and those formed endogenously under conditions of hyperglycemia and oxidative stress act synergistically to increase the overall body burden of AGEs. A proportion of dietary AGEs is absorbed through the gastrointestinal tract and can accumulate in tissues, whereas hyperglycemia promotes the endogenous generation of highly reactive dicarbonyl compounds, such as glyoxal and methylglyoxal, which serve as direct precursors of AGEs, including CML and CEL [29]. The accumulation of AGEs contributes to tissue damage through two major mechanisms: direct biochemical modification of proteins, and activation of cellular signaling pathways via receptors such as RAGE (Figure 2). These complementary processes generate a self-amplifying cycle of oxidative stress, inflammation, and tissue dysfunction that is highly relevant to CKM syndrome [30].

1. Structural modification of proteins. AGEs irreversibly modify extracellular matrix proteins such as collagen and elastin, promoting cross-linking, stiffening, and loss of elasticity. These alterations impair vascular compliance, increase pulse-wave velocity, and contribute to hypertension and microvascular injury. AGE-mediated cross-linking also disrupts matrix turnover by reducing proteolytic susceptibility, accelerating the accumulation of dysfunctional matrix proteins in vascular, renal, and cardiac tissues. These changes are particularly pronounced for AGEs such as CML, CEL, and pentosidine, which accumulate under chronic hyperglycemia, oxidative stress, and high dietary AGE intake [31].

2. Activation of RAGE signaling. The second major mechanism involves binding of AGEs to RAGE, a multiligand pattern-recognition receptor expressed on endothelial cells, smooth muscle cells, immune cells, and adipocytes. Engagement of RAGE initiates a broad inflammatory and oxidative signaling program: AGE–RAGE interaction activates NADPH oxidase, driving reactive oxygen species (ROS) production, and stimulates transcription factors including NF-κB, MAPKs, and JAK/STAT [31,32]. Key downstream consequences include:

- Amplification of oxidative stress through NADPH oxidase activation and mitochondrial dysfunction.
- NF-κB-driven inflammation, with increased expression of TNF-α, IL-6, IL-1β, VCAM-1, and ICAM-1.
- Endothelial dysfunction via reduced nitric oxide bioavailability and eNOS uncoupling.
- Enhanced leukocyte adhesion and vascular infiltration, promoting atherosclerotic plaque formation.
- Positive-feedback upregulation of RAGE, sustaining chronic inflammatory activation.

This self-reinforcing loop is central to the progression of vascular injury, insulin resistance, and metabolic dysfunction.

3. Metabolic consequences: insulin resistance and adipose dysfunction. AGE–RAGE signaling interferes with insulin receptor signaling by activating stress kinases (e.g., JNK, p38 MAPK) that inhibit IRS-1 phosphorylation and downstream PI3K–Akt signaling, leading to impaired glucose uptake, increased insulin resistance, and adipose tissue dysfunction. In adipose tissue, AGEs promote macrophage infiltration, reduce adiponectin secretion, and impair lipid handling, contributing to systemic insulin resistance and dyslipidemia [32].

4. An integrated toxicity model. Together, structural protein modification and RAGE-mediated signaling create a biochemical environment characterized by oxidative stress, chronic inflammation, endothelial dysfunction, and impaired metabolic regulation. These mechanisms provide a biologically plausible framework through which high dietary AGE intake (from UPFs as well as from other foods prepared with dry heat) could contribute to the development and progression of diabetes, cardiovascular disease, kidney injury, and the broader CKM syndrome, although the relative contribution of dietary versus endogenously formed AGEs remains to be established in humans.

## 3. Food Neo-Contaminants Beyond AGEs

In addition to AGEs, thermal processing generates a diverse group of chemical contaminants—collectively termed neo-contaminants—that arise from Maillard chemistry, sugar dehydration, amino acid pyrolysis, and lipid oxidation. Neo-contaminants formed during cooking and industrial thermal processing include acrylamide, furan, 5-hydroxymethylfurfural (HMF), heterocyclic amines (HCAs), polycyclic aromatic hydrocarbons (PAHs), and chloropropanols such as 3-MCPD and glycidol. These compounds have traditionally been studied for carcinogenic potential, but accumulating evidence indicates that several also exert metabolic, endocrine, and inflammatory effects highly relevant to the toxicity profile of UPFs [33].

**Figure 3:**
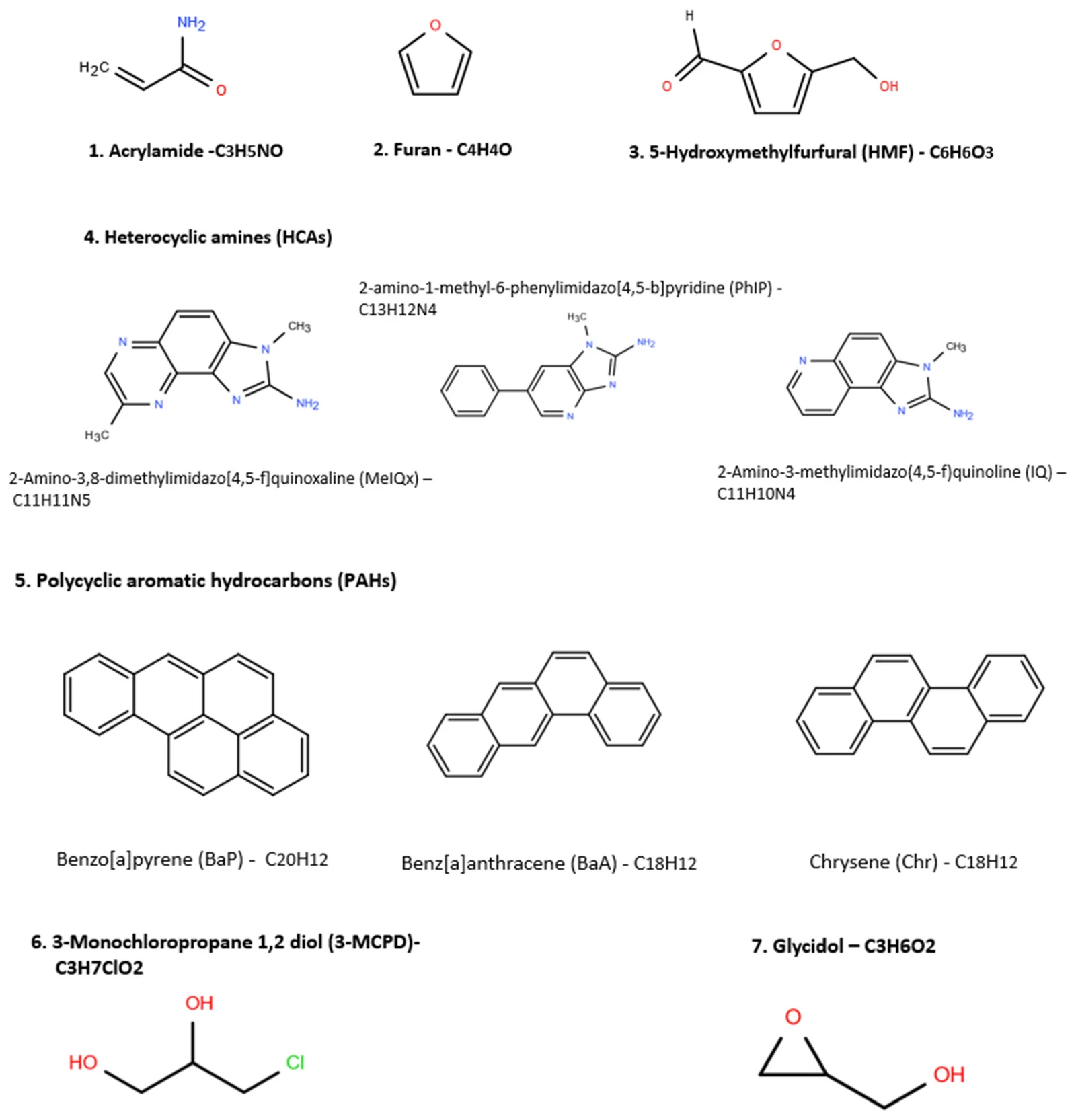
Chemical structures of several neo-contaminants of interest found in ultra-processed foods: (**1**) acrylamide; (**2**) furan; (**3**) 5-hydroxymethylfurfural; (**4**) heterocyclic amines; (**5**) polycyclic aromatic hydrocarbons; (**6**) 3-monochloropropane-1,2-diol; and (**7**) glycidol.

A unifying feature of these contaminants is their co-generation with Maillard-derived flavor compounds. Highly browned foods—fried snacks, roasted coffee, grilled meats—produce attractive aromas that activate reward pathways and may help explain the potentially addictive appeal of high-AGE, highly processed foods. This sensory reinforcement may amplify exposure to neo-contaminants while simultaneously driving excess caloric intake [34]. Figure 3 shows the chemical structures of several neo-contaminants of interest.

1. Acrylamide. Acrylamide forms predominantly through the Maillard reaction between asparagine and reducing sugars during high-temperature, low-moisture processing (>120 °C) [35]. It is classified as a probable human carcinogen, but its toxicological profile extends beyond carcinogenicity [36]. Acrylamide and its metabolite glycidamide induce oxidative stress, neurotoxicity, reproductive toxicity, and DNA adduct formation. Importantly, emerging evidence identifies acrylamide as a metabolic disruptor that promotes adipogenesis, dysregulates lipid and glucose metabolism, and exacerbates insulin resistance—effects that are particularly pronounced in individuals with diabetes [37]. These findings position acrylamide as a plausible obesogenic, linking UPF consumption to metabolic disease through endocrine-disrupting pathways [38].

2. Furan. Furan is a highly volatile compound generated from the thermal degradation of carbohydrates, amino acids, polyunsaturated lipids, carotenoids, and ascorbic acid. It is abundant in roasted coffee, jarred baby foods, canned products, and cereal-based snacks [39,40,41,42,43]. Classified as a possible human carcinogen, furan undergoes metabolic activation by CYP2E1 to form cis-2-butene-1,4-dial (BDA), a reactive electrophile that forms adduct with proteins and nucleic acids. Furan exposure induces hepatotoxicity, nephrotoxicity, oxidative stress, inflammation, and epigenetic alterations [44]. Margin-of-exposure analyses consistently indicate potential public health concern, especially for infants consuming thermally processed baby foods.

3. 5-Hydroxymethylfurfural (HMF). HMF arises from sugar dehydration and Maillard reactions in carbohydrate-rich foods such as baked goods, coffee, honey, fruit juices, and dairy products [45,46,47]. Although less acutely toxic than acrylamide or furan, HMF can convert to reactive derivatives—including chloromethylfurfural and sulfoxymethylfurfural—that exhibit mutagenic and cytotoxic properties. High HMF concentrations have been associated with mucosal irritation, oxidative stress, and potential contributions to neurodegenerative, cardiovascular, and metabolic disorders [45,46,47]. Notably, correlations between HMF and acrylamide levels in fried and baked foods suggest that HMF may serve as a practical indicator of acrylamide formation in industrial processing [48,49].

4. Heterocyclic amines (HCAs). HCAs form during high-temperature cooking of protein-rich foods, particularly meats and seafood, through reactions involving amino acids, creatine/creatinine, and sugars. They fall into two major categories [50]:

- Pyrolytic aminocarbolines (ACs), generated at >300 °C (grilling, roasting, frying);
- Amino-imidazo-azaarenes (AIAs), formed at 100–300 °C (pan-frying, baking).

Several HCAs—including MeIQx, PhIP, Glu-P-1, and IQ—are classified as Group 2A/2B carcinogens. Beyond mutagenicity, HCAs promote oxidative stress, inflammation, and endocrine disruption. Chronic exposure has been linked to risk of colorectal, breast, and prostate cancers, and emerging evidence suggests that HCAs may impair metabolic homeostasis through mitochondrial stress and inflammatory signaling.

5. Polycyclic aromatic hydrocarbons (PAHs). PAHs form during incomplete combustion of organic matter, particularly in charred or smoked foods. Several PAHs (e.g., benzo[a]pyrene) are potent carcinogens and endocrine disruptors. Their lipophilicity facilitates bioaccumulation in adipose tissue, where they may contribute to chronic inflammation and metabolic dysfunction [51].

6. 3-MCPD and glycidol. Chloropropanols such as 3-monochloropropane-1,2-diol (3-MCPD) and glycidol form during high-temperature refining of vegetable oils and in baked or fried foods containing fats. Glycidol is highly genotoxic, forming DNA adducts and inducing heritable mutations. Both compounds impair reproductive function, disrupt steroidogenesis, and induce oxidative stress. Given the widespread use of refined oils in UPFs, chloropropanol exposure may represent an underappreciated metabolic risk [52].

### 3.1. Integrated Perspective

Although historically studied in isolation, these neo-contaminants share several mechanistic features with one another and with AGEs:

- Generation under high-temperature, low-moisture conditions common in, but not exclusive to, the industrial processing of many UPFs;
- Promotion of oxidative stress and inflammation;
- Endocrine disruption and metabolic impairment;
- Co-occurrence in the same foods, raising the possibility of combined (additive or interactive) effects, which remain largely unexplored experimentally;
- Coupling with reward-enhancing Maillard aromas that drive overconsumption.

Collectively, these compounds form a chemical signature of UPFs that extends far beyond macronutrient composition. Their combined biological effects provide a plausible mechanistic framework that may help explain the associations of UPF consumption with obesity, insulin resistance, diabetes, cardiovascular disease, and CKM syndrome [33]. Rasool et al. proposed that acrylamide and advanced glycation end products (AGEs), which are simultaneously generated during high-temperature processing and frequently coexist in fried foods, may exert additive or synergistic toxic effects. The authors suggest that this interaction is mediated through shared mechanisms involving glutathione depletion, oxidative stress, reactive oxygen species generation, inflammation, and activation of the AGE-RAGE signaling pathway. However, direct experimental studies specifically evaluating the combined toxicity of acrylamide and AGEs remain scarce, and current evidence is largely based on mechanistic convergence rather than controlled co-exposure models [53]. Although direct evidence of synergistic toxicity among food contaminants remains limited, several contaminants converge on common molecular pathways, including oxidative stress, inflammation, mitochondrial dysfunction, genotoxicity, and apoptosis. Therefore, simultaneous exposure to multiple contaminants may enhance biological damage through additive or synergistic interactions, resulting in a greater overall toxic burden than exposure to individual compounds alone [54]. This framework, however, still requires confirmation in human studies that take into account the co-occurrence of these compounds in the same foods.

### 3.2. Human Dietary Exposure and Risk Assessment of AGEs and Food Neo-Contaminants

Unlike the neo-contaminants discussed above, dietary AGEs have not been the subject of a formal risk assessment by food safety agencies, and no health-based guidance value has been established. Estimates of usual intake depend strongly on the analytical method underlying the food composition database. Using the ELISA-based database, the mean intake of a healthy adult population in New York was estimated as approximately 15,000 kU AGE/day [17], whereas databases based on UPLC–MS/MS indicate mean intakes of approximately 2.4 mg/day each of protein-bound CML and CEL and approximately 28 mg/day of MG-H1 (energy-adjusted) in Dutch adults aged 45 years and older (mean age 67 years) [55,56,57,58,59,60]. Major dietary sources include meat cooked with dry heat, high-fat and processed cheeses, fried foods, and baked cereal products, many of which are UPFs [17,61]. Data in children remain scarce; however, because of their higher food intake per kilogram of body weight and their frequent consumption of thermally processed products such as infant formula, breakfast cereals, and biscuits, body-weight-adjusted exposure is expected to be higher than in adults [62].

In the Italian I. Family cohort, dietary AGE intake varied considerably among children and adolescents, ranging from approximately 6900 kU/day in the lowest tertile to more than 28,400 kU/day in the highest tertile. Participants with the highest AGE intake exhibited dietary patterns characterized by greater consumption of protein- and fat-rich foods, particularly meat products [63].

For acrylamide, EFSA estimated average dietary exposures of 0.4–1.9 µg/kg bw/day and high exposures of 0.6–3.4 µg/kg bw/day, with infants, toddlers, and children showing the highest intake [9]. Main sources included fried potato products, bread, cereals, biscuits, and coffee, depending on age group. Home frying practices may increase exposure by up to 80% [9]. Although non-neoplastic effects were not considered a concern, the calculated MOEs for carcinogenicity were well below 10,000, indicating a potential health concern [9]. This evidence led to mandatory mitigation measures and benchmark levels in the European Union [59].

For furan, infants were the most exposed group through ready-to-eat meals, while grain products and coffee were the main sources in older age groups [56]. MOE estimates based on a BMDL10 of 0.064 mg/kg bw/day indicated a health concern, supporting continued monitoring of furan and alkylfurans in foods [56,64].

Exposure to 3-MCPD and glycidol, mainly derived from refined vegetable oils, was estimated at 0.2–1.5 and 0.1–0.9 µg/kg bw/day, respectively, with the highest intake in infants and young children [57]. EFSA established a TDI of 2 µg/kg bw/day for 3-MCPD, which may be exceeded by high-consuming young children and formula-fed infants [58]. Glycidol remains of concern because of its genotoxic and carcinogenic properties, with MOEs below the level considered of low concern [57]. Consequently, maximum levels have been established in oils and infant formula [57].

For HMF, no health-based guidance value is available, although coffee, dried fruits, honey, and baked products are major sources [45]. For PAHs, EFSA considered average dietary exposure to be of low concern, but potential concern remains for high consumers [60]. HCAs are primarily associated with well-done meat and fish, although no harmonized international risk assessment is currently available [50].

Overall, infants and young children consistently represent the most exposed population groups. However, current risk assessments evaluate contaminants individually, and the health implications of combined exposure to AGEs and multiple neo-contaminants present in the same thermally processed foods remain largely unknown [9,56,57,60].

## 4. Dietary AGEs and Food Neo-Contaminants as Potential Contributors to UPF Toxicity

Food composition and processing are complex, and multiple mechanisms—acting independently or together—may explain the adverse health effects of UPFs: food additives, high energy density, altered food matrices, poor nutritional quality, contaminants generated during thermal processing, gut microbiota dysbiosis, and increased intestinal permeability [65]. AGE generation during industrial processing and home cooking satisfies several criteria supporting a contributory role of AGEs in the adverse effects of UPFs [30]:

1. AGEs are generated in food during thermal treatment, whether industrial or domestic.

2. Specific food-derived AGEs, such as CML, are structurally and physiologically indistinguishable from their endogenous counterparts.

3. Specific AGEs, including CML, whether endogenous or exogenous, have been shown to injure multiple cell systems and tissues.

4. Oral administration of labeled AGE loads in humans demonstrates absorption into the circulation, with most eliminated in urine and some retained in the circulation and tissues.

5. AGE-enriched diets induce insulin resistance or type 2 diabetes in mouse models, whereas low-AGE diets prevent these outcomes [66,67].

6. Healthy human subjects consuming AGE-enriched diets show increased inflammatory and insulin-resistance markers [68].

7. Individuals with insulin resistance or type 2 diabetes who consume AGE-enriched diets show increased inflammatory and insulin-resistance markers, whereas low-AGE diets exert the opposite effect [69,70].

Taken together, the temporal relationship between AGE exposure and insulin resistance, along with their covariation, supports a plausible causal relationship.

Substantial evidence links AGEs with insulin resistance and diabetes. The food neo-contaminants discussed here have been most strongly associated with carcinogenicity and hepatotoxicity, whereas evidence for their metabolic effects is less consistent. Nevertheless, several of these compounds may contribute to insulin resistance, β-cell dysfunction, obesity, and ultimately type 2 diabetes. Because the strength of evidence varies considerably across compounds, compound-specific mechanistic and translational studies are needed. Moreover, because AGEs and neo-contaminants are generated by the same reactions and under the same processing conditions, they co-occur in the same foods (Table 1), and their individual contributions are difficult to disentangle in human studies; estimates of dietary AGE intake may therefore partly act as markers of the overall burden of thermally generated compounds, and vice versa.

Table 1 presents the reported contents of CML and CEL in commonly consumed ultra-processed foods, along with information on food origin or cooking method and analytical determination techniques; to facilitate comparison across studies, all concentrations were converted to a common unit (mg/g food). Banana is included as a minimally processed reference food. CML and CEL values for beef correspond to baked beef burger slices [71] and were converted from mg/kg protein to mg/100 g food values as reported in that study.

Table 2 presents the reported contents of neocontaminants (acrylamide, furan, and 5-HMF) in food products commonly classified as ultra-processed foods. All values were converted to a common unit, µg/kg.

**Figure 4:**
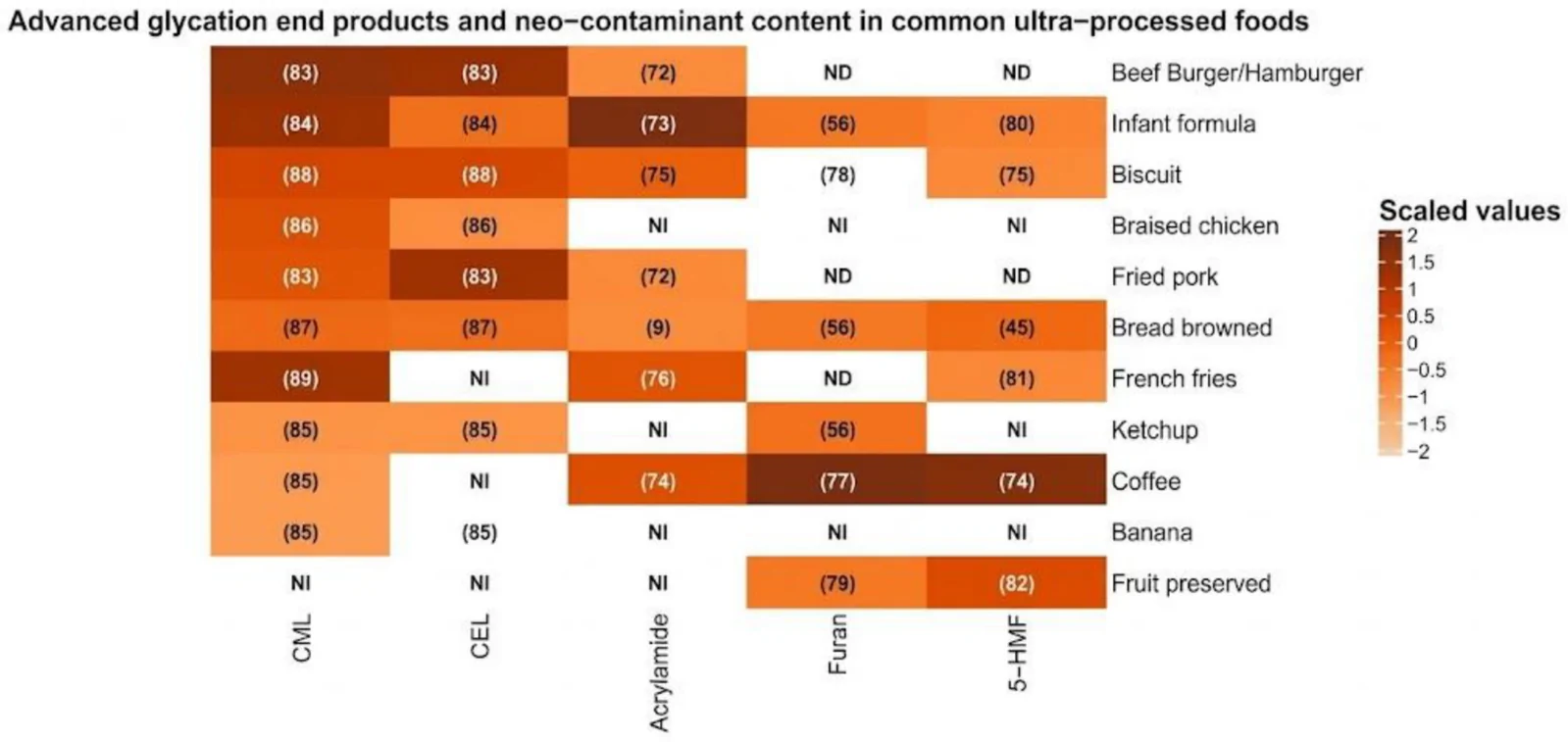
Heat map showing harmonized concentrations (μg/kg) of advanced glycation end products (CML and CEL) and neo-contaminants (acrylamide, furan, and 5-HMF) in selected ultra-processed foods. Foods are arranged from highest to lowest CML content. Each row represents a food item and each column represents a contaminant; color intensity reflects scaled concentration values, with darker colors indicating higher relative levels. Because contaminant concentrations spanned several orders of magnitude, data were log_10_-transformed after adding 1 to avoid issues associated with zero values. The transformed data was subsequently standardized by neo-contaminant using z-scores to facilitate comparison among food products. The scale shown on the right corresponds to standardized values (z-scores), where 0 represents the average value, positive values (up to +2) above the average, and negative values (up to −1.5) below the average. NI = no information available; ND = not detected. CML = Nε-(Carboxymethyl)lysinE; CEL = Nε-(Carboxyethyl)lysine; 5-HMF = 5-Hydroxymethylfurfural. Numbers in parentheses denote the bibliographic source of the reported concentration.

Figure 4 shows the heatmap that visually summarizes the concentrations of advanced glycation end products (AGEs) and food-processing contaminants in consumed ultra-processed foods. Values for Nε-carboxymethyllysine, Nε-carboxyethyllysine, acrylamide, furan, and 5-hydroxymethylfurfural are reported in Table 1 and Table 2, extracted from published studies [9,45,56,71,72,73,74,75,76,77,78,79,80,81,82,83,84,85,86,87,88].

Limitations should be considered when comparing the concentrations reported in Table 1 and Table 2 and Figure 4. The values were obtained from different studies using diverse analytical methodologies, including LC-MS/MS, UPLC-MS/MS, ELISA, HPLC, HPLC-MS/MS, GC-MS, and LC-ESI-MS/MS, methods, each with distinct sensitivity, specificity, extraction procedures, calibration strategies, and limits of detection. In the case of AGEs, differences between immunochemical methods and mass spectrometry-based techniques are particularly relevant, as they may quantify different AGE fractions and yield substantially different concentration estimates. In addition, variations in sample preparation, food matrix characteristics, and reporting units may further affect comparability. Therefore, the values presented should be interpreted as indicative of the relative occurrence of AGEs and neo-contaminants in different foods rather than as directly comparable absolute concentrations. Although direct quantitative comparisons across studies are limited by methodological heterogeneity, the review is valuable because it consolidates dispersed information on AGEs and neo-contaminants in ultra-processed foods, identifies major food sources and exposure trends, and highlights critical gaps in the current literature. By bringing together this data, the review provides a useful framework for future research.

## 5. Strategies to Reduce Formation of AGEs and Other Neo-Contaminants During Processing and Cooking

### 5.1. Cooking Methods That Reduce AGE Formation

A substantial body of evidence indicates that dietary AGE exposure is influenced more strongly by cooking method than by the intrinsic composition of raw foods. This largely reflects activation of the Maillard reaction, a non-enzymatic process involving reducing sugars and proteins or lipids that depends strongly on heat, time, and moisture [17,89].

Dry-heat methods—grilling, baking, roasting, and frying—are consistently associated with markedly increased AGE formation. These techniques can raise AGE levels several-fold relative to raw foods, primarily because they involve high temperatures (>140 °C), low water activity, and enhanced lipid oxidation [17,61]. Under these conditions, well-characterized AGE compounds such as CML and MG-H1 form in substantial amounts, as elevated temperature and reduced moisture promote advanced stages of the Maillard reaction and accelerate protein modification and oxidative processes [19,61]. Frying is among the most potent promoters of AGE accumulation because high temperatures, lipid-rich environments, and prolonged cooking times enhance both glycation reactions and the formation of additional harmful compounds, including acrylamide [53]. Protein- and fat-rich foods, such as meats and cheeses, are especially susceptible to AGE formation during dry-heat cooking because of their high content of reactive amino groups and lipid-oxidation products [17].

Moist-heat methods—boiling, steaming, poaching, and low-temperature water-based cooking—are associated with substantially lower AGE formation, primarily because of lower temperatures (typically around 100 °C), higher moisture content, and reduced oxidative conditions [90]. Water limits the Maillard reaction by diluting reactants and reducing thermal degradation, thereby inhibiting AGE formation; as a result, moist-heat methods generally produce substantially lower AGE concentrations than dry-heat techniques [89,90].

### 5.2. Emerging Technologies to Reduce AGEs in UPFs

Several emerging technologies have been proposed for mitigating AGE formation in UPFs [33], including optimized thermal approaches such as low-temperature vacuum heating [91], vacuum microwave and infrared heating, and non-thermal technologies such as high-pressure processing [92], pulsed electric fields [93], ultrasound [85], and cold plasma [94]. However, the strength of the supporting evidence differs considerably among them, and most still require validation in industrial food production [33]. Direct evidence of reduced AGE formation in real foods is available for only a few: compared with conventional processing, microwave roasting reduced free CML in hazelnuts by approximately 63%, air frying reduced free and protein-bound CML and CEL in chicken breast relative to deep-frying, and high pressure combined with moderate heat pre-incubation reduced protein-bound CML and CEL in milk by approximately 75% and 82% relative to pasteurization and ultra-high-temperature treatment, respectively [33]. Results are not uniformly favorable: air-fried cupcakes contained more AGEs than oven-baked ones, and low-temperature vacuum heating increased free CML and CEL in sturgeon fillets compared with boiling, probably because of the longer heating time [33]. For other technologies, the evidence in real foods concerns mainly acrylamide and HMF rather than AGEs: vacuum frying reduced acrylamide and furan in potato chips by approximately 58% and 81%, respectively, compared with atmospheric frying [95], and pretreatments such as ultrasound-assisted leaching [48,85] and pulsed electric fields [96] reduce acrylamide (and HMF) by promoting the removal of reducing sugars and asparagine. Evidence on AGEs for pulsed electric fields and cold plasma derives from protein–sugar model systems and is inconsistent; cold plasma, for instance, can increase protein oxidation and carbonylation, which may favor AGE formation [33]. Moreover, several of the studies cited for these technologies address general effects on food quality, microstructure, or nutritional value rather than AGEs [91,92,93,94]. Because AGEs and other neo-contaminants share precursors and formation conditions, similar benefits are plausible, but studies quantifying individual AGEs (e.g., CML, CEL, and MG-H1) by LC–MS/MS in industrially relevant foods are needed. Nanomaterials have also been proposed to inhibit AGE formation; however, uncertainties regarding the toxicity, migration, and regulatory status of such materials in foods [97] mean that they cannot currently be regarded as a practical or safe strategy for reducing AGEs in UPFs; the anti-glycation activity reported for metal nanoparticles (e.g., silver nanoparticles) is derived from in vitro studies [98].

### 5.3. Exogenous Additives

Functional additives such as food gels are gaining attention for their ability to enhance the stability of bioactive compounds like polyphenols during processing, which may help reduce AGE formation [99,100]. Natural antioxidants have also shown potential to inhibit glycation pathways and scavenge reactive intermediates involved in AGE generation [101]. A recent review highlights encapsulated polyphenols and other bioactive-compound protection systems as promising strategies for reducing AGE formation during thermal processing [102]. However, few studies simulate AGE formation during thermal processing, and fewer still use real food systems. Wang et al. therefore suggested that future research incorporate high-temperature models—such as lysine-reducing sugar and arginine-reducing sugar systems—focusing on the stability of these additives and their inhibitory effects on AGEs [33].

### 5.4. Reversal of Glycation: Is It Possible?

AGEs have long been regarded as irreversible products of protein aging, but recent findings challenge this paradigm. Trabosh et al. demonstrated that CML can be enzymatically removed from proteins and aged human tissues using an engineered enzyme (CMLase), suggesting that at least some forms of glycation-derived protein damage may be reversible. Further studies are required to determine whether such molecular repair is feasible and whether it translates into functional recovery in vivo [103].

## 6. Conclusions

The available evidence is consistent with the view that thermal processing may contribute to the adverse health profile of many UPFs, alongside their formulation, energy density, additives, and nutritional quality, by promoting the formation of AGEs and multiple food neo-contaminants with inflammatory, oxidative, metabolic, and toxicological effects. Although the strength of evidence differs across compounds, the convergence of mechanistic, experimental, and human data suggests that reducing exposure to these processing-derived products may represent an important strategy for lowering cardiometabolic risk.

Future research should refine exposure assessment, identify the most biologically relevant compounds and food sources, clarify dose–response relationships, and evaluate whether practical changes in food processing, product formulation, and cooking practices can reduce AGE and neo-contaminant burden without compromising food safety, nutrition, or acceptability.

## Figures and Tables

**Table 1:** Contents of advanced glycation end products in common ultra-processed foods.

Food Product	Obtained from/Cooking Method	CML Content mg/100 g Food	CEL Content mg/100 g Food	Detection Method	Reference
Beef burger	Dried meat baked	13.37 ± 2.28	8.39 ± 0.79	LC-MS/MS	[71]
Infant formula	Purchased from supermarket	8.42 ± 0.28	0.234 ± 0.021	UPLC-MS/MS	[72]
Biscuit	Made with hulless oat cereal (7 min)	2.14 ± 0.26	1.54 ± 0.07	LC-MS/MS	[73]
Braised chicken	Did not provide detailed cooking information	1.169–1.612	0.027–0.038	ELISA	[74]
Fried pork	Obtained from supermarket, did not provide detailed cooking information	1.09 ± 0.09	6.01 ± 0.26	UPLC-MS/MS	[71]
Bread browned	Obtained from industrial production	0.49 ± 0.15	0.25 ± 0.002	HPLC-MS/MS	[75]
French fries	Potatoes frying	0.13	NI	UPLC-MS/MS	[76]
Ketchup	Obtained from supermarket	0.05	0.01	UPLC-MS/MS	[77]
Coffee	Obtained from supermarket	0.014	NI	ELISA	[77]
Banana	Obtained from supermarket	0.01	0	UPLC-MS/MS	[77]

The data are presented as mean or mean ± SD (standard deviation). NI: no information; CML: Nε-(carboxymethyl)lysine); CEL: Nε-(carboxyethyl)lysine).

**Table 2:** Contents of neo-contaminants in common ultra-processed foods.

Food Product	Obtained from/Cooking Method	Acrylamide µg/kg	Detection Method	Furan µg/kg	Detection Method	5-HMF µg/kg	Detection Method
Hamburger	Frying foodstuffs purchased, prepared in laboratory under standardized conditions.	14 [78]	GC-MS LC-MS/MS	ND		ND	
Infant formula	Commercial milk-based powdered infant formula.	1821 [79]	GC/MS	10.5 [56]	Reported by EFSA Panel	4170–21,410 [80]	RP-HPLC-DAD
Instant Coffee	Standardized in vitro gastrointestinal digestion protocol (INFOGEST) was used. Not for furan.	589 [81]	LC-ESI-MS/MS	1700–3500 [82]	Headspace-GC-MS	2,890,000 [81]	LC-DAD
Bread browned	Commercial white bread, toasted for 4 min.	31 [9]	HPLC	<2–18 [56]	Reported by EFSA Panel	11,800–87,700 [45]	HPLC-UV/DAD
Biscuit	Refined wheat flour biscuits, 200 °C for 10 min, mean from 2 samples.	355 [83]	LC-ESI-MS-MS	0.0324 [84]	HS GC-MS with isotope dilution.	5350–13,200 [83]	HPLC
Ketchup		NI		101.6 [56]	Reported by EFSA Panel	NI	
French fries	Samples by triplicate, prepared under standardized laboratory conditions [12].	502.7 ± 52.3 [85]	LC-MS/MS	ND		8430 ± 160 [86]	UV spectrophotometer
Fried pork	Homogenization, centrifugation and ultrafiltration.	52 [78]	LC-MS/MS	ND		ND	
Fruit preserved	Canned *.			7 * [87]	Reported by EFSA	30,000–280,000 [88]	RP-HPLC UV-detector

NI: no information; ND: not detected; 5-HMF: 5-Hydroxymethylfurfural. * Product canned.

## Data Availability

No new data were created or analyzed in this study. Data sharing is not applicable to this article.
